# A Shared-Electrode and Nested-Tube Structure Triboelectric Nanogenerator for Motion Energy Harvesting

**DOI:** 10.3390/mi10100656

**Published:** 2019-09-29

**Authors:** Zhumei Tian, Guicheng Shao, Qiong Zhang, Yanan Geng, Xi Chen

**Affiliations:** 1 Department of Electronics, Xinzhou Teachers University, Xinzhou 034000, China; xzsfxysgc@163.com (G.S.); zq2019zhangqiong@163.com (Q.Z.); gynblue@163.com (Y.G.); 2 Science and Technology on Electronic Test and Measurement Laboratory, North University of China, Taiyuan 030051, China; nanochenxi@163.com

**Keywords:** triboelectric nanogenerator, electrification, shared-electrode, energy harvesting

## Abstract

Triboelectric nanogenerators with the function of harvesting human motion energy have attracted wide attention. Here, we demonstrate a shared-electrode and nested-tube structure triboelectric nanogenerator (SNTN) for harvesting human motion energy. The design of the SNTN employs flexible silicone rubber as the negative friction material and Ni-coated polyester conductive textile as the positive friction material and the electrode material. The entire structure consists of an inner triboelectric unit and an outer triboelectric unit. The inner triboelectric unit is formed by a hollow inner tube and a hollow middle tube, while the hollow middle tube and a hollow outer tube constitute the outer triboelectric unit. The hollow middle tube is used as the shared tube, and the electrode in the middle tube is used as the shared electrode of the two triboelectric units. Our research demonstrates that the output performance of the SNTN was improved significantly compared with a single triboelectric unit due to the cooperation of the two triboelectric units. When the SNTN is pressed by 300 N external force, output open-circuit voltage of 180 V and output short-circuit current of 8.5 μA can be obtained. The output electrical energy can light up 31 light-emitting diodes (LEDs) connected serially (displaying “XZTC”) and can drive a digital clock after rectifying storage, which shows application prospects in the field of illuminating devices and portable electronics.

## 1. Introduction

As a novel energy harvesting method, triboelectric nanogenerators (TENGs), whose theoretical sources are derived from Maxwell displacement current [1], have been proposed to convert wave energy [2], wind energy [3], rain power [4,5], vibration energy [6,7,8], and human motion energy [9,10,11,12,13,14] into electricity as a result of the coupling of triboelectrification and electrostatic induction [3,4,15,16,17,18,19,20]. TENGs have the advantages of low cost, small size, and high output performance, and they have great application prospects in portable electronics [5,17,21,22,23], biomedical microsystems [24,25], healthcare [23,26], sensor applications [27,28,29], and so forth.

With the widespread adoption of flexible wearable electronic devices, many researchers are working on flexible wearable triboelectric nanogenerators for human motion energy harvesting which are expected to be flexible, stretchable, environmentally friendly, and sustainable. Triboelectric nanogenerators for human motion energy harvesting have a fabric structure, tube structure, or fiber structure; among these, the triboelectric nanogenerators with a fabric structure are mainly used for human motion energy harvesting [11,30,31,32], while the fiber-based and tube-based triboelectric nanogenerators can be adapted for human motion energy harvesting and human motion information monitoring due to their flexibility and direction adaptability [12,13,33].

Herein, we designed a shared-electrode and nested-tube structure triboelectric nanogenerator (SNTN) for harvesting human motion energy. The whole structure consists of a flexible hollow inner tube, a hollow middle tube, and a hollow outer tube. The inner triboelectric unit is formed by nesting the hollow inner tube in the hollow middle tube, and the outer triboelectric unit is formed by nesting the hollow middle tube in the hollow outer tube; the two triboelectric units share the hollow middle tube and the electrode layer in the middle tube as a shared electrode. The airbag is formed in the gap between the inner tube, the middle tube, and the outer tube. The structure of the SNTN is encapsulated by silicone rubber to protect the device from ambient contamination. The prepared SNTN has the characteristics of flexibility, sustainability, biological compatibility, etc., and can convert energy from different pressing directions perpendicular to the axial direction into electrical energy.

## 2. Materials and Methods

### 2.1. Material Preparation

Through uniformly mixing silicone and curing agent at a 50:1 weight ratio, then leaving the mixture at room temperature for 6 h, silicone rubber with good flexibility and a strong tendency to gain electrons was prepared [32].

### 2.2. Fabrication of the SNTN

**Figure 1:**
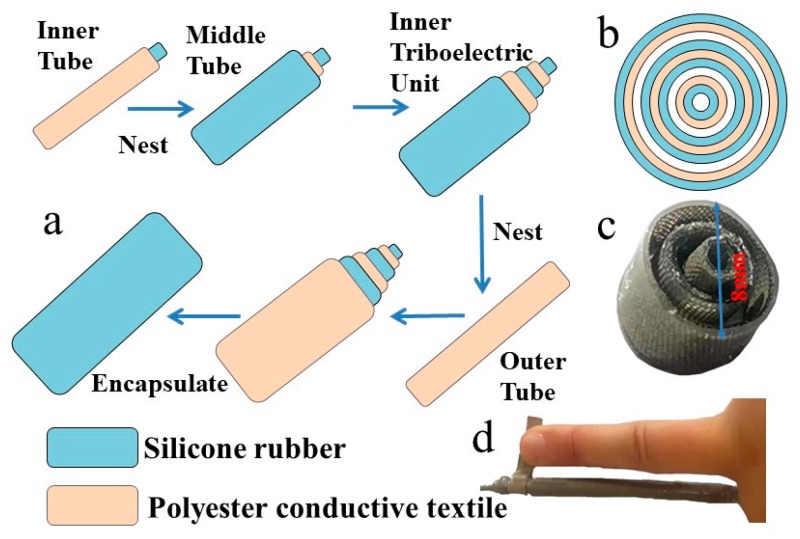
A structural schematic of the shared-electrode and nested-tube structure triboelectric nanogenerator (SNTN): (**a**) The fabrication processes of the SNTN; (**b**) A cross-sectional schematic diagram of the SNTN; (**c**) A cross-sectional image of the fabricated SNTN. (**d**) A comparison photo between the fabricated SNTN and the middle finger.

The structure and fabrication processes of the SNTN are illustrated in Figure 1. The structure employs flexible silicone rubber as the negative friction material and Ni-coated polyester conductive textile as the positive friction material and the electrode material. Scanning electron microscope (SEM) showed that the surface of the Ni-coated polyester conductive textile has microstructure, which can effectively improve the output performance of the triboelectric nanogenerator (Figure S1). The whole structure was composed of a hollow inner tube, a hollow middle tube, and a hollow outer tube. The hollow silicone tube in the inner tube was used as the structural base of the hollow inner tube, and the outer surface of the silicone tube was coated with Ni-coated polyester conductive textile as the positive friction material and the inner electrode of the inner triboelectric unit. The hollow middle tube was composed of three parts: an inner dielectric layer as the negative friction material of the inner triboelectric unit, a shared electrode layer, and an outer dielectric layer as the negative friction material of the outer triboelectric unit. The hollow Ni-coated polyester conductive textile outer tube was used as the positive friction material of the outer triboelectric unit and the outer electrode. The hollow inner tube with a smaller diameter was nested in the hollow middle tube, then nested in the hollow outer tube with the largest diameter, then the nested structure was entirely encapsulated using the prepared silicone rubber mixture. The airbag was formed in the gap between the inner tube, the middle tube, and the outer tube. By following the above operation procedures, an SNTN was prepared successfully.

### 2.3. Electrical Measurement of the SNTN

The surface morphology of the Ni-coated polyester conductive textile was tested by SEM (ZEISS EVO18, Carl Zeiss, Jena, Germany). The output performance of the prepared SNTN could be measured by a linear motor (OuBang, Taizhou, China) to simulate the press–release process, and a keithley 6514 (Tektronix U.K. Limited, Berkshire, UK) was used to test the output short-circuit current and open-circuit voltage. The pressure information of the measurement process could be detected using a pressure sensor (QLMH-P) and a high-speed response display instrument (QL-8016) (QL Sensors Company, Anhui, China).

## 3. Results and Discussion

### 3.1. Working Principle of the SNTN

**Figure 2:**
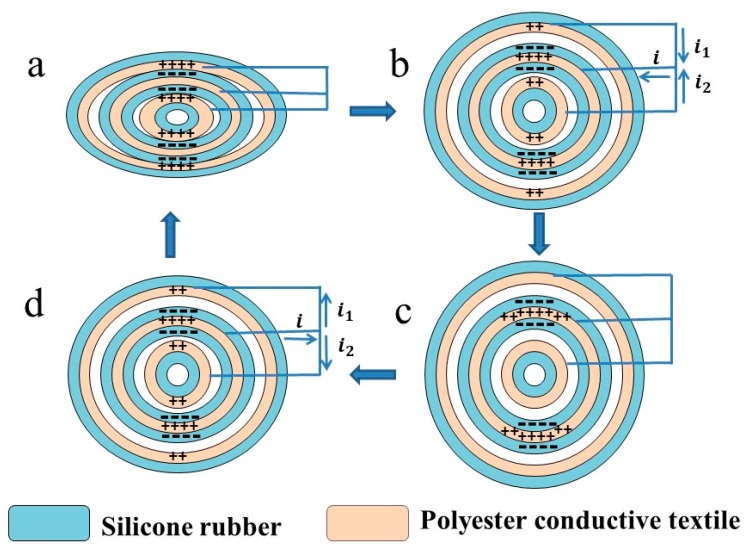
Schematic illustration of the power generation mechanism of the SNTN. Triboelectric charge distribution of one current generation cycle: (**a**) full-contact state; (**b**) releasing state; (**c**) full-separation state (initial state); (**d**) pressing state.

The power generation mechanism of the SNTN is illustrated in Figure 2. Because silicone rubber and Ni-coated polyester conductive textile have different electron affinity potential energy, here, silicone rubber was used as a negative friction materials and Ni-coated polyester conductive textile was used as a positive friction material [34]. There was no charge transferred in the initial state where the positive friction material and the negative friction material in the inner triboelectric unit and the outer triboelectric unit were separated. When an external force, such as pressing or bending, is applied on the SNTN, structural deformation will happen. The positive friction material and the negative friction material of the inner triboelectric unit will come into contact with each other, and the negative friction material and the positive friction material of the outer triboelectric unit will come into contact with each other, too; then the negative friction material will be charged negatively and the positive friction material will be charged positively (Figure 2a). When the external force is revoked, the contact surfaces of the two triboelectric units will separate due to the existence of the airbag and the elasticity of the silicone rubber. They then form an electric potential difference, which causes electrons to flow from the shared electrode layer in the middle tube to the inner electrode of the inner tube and the outer electrode of the outer tube through the external circuit to balance the generated triboelectric electric potential (Figure 2b). When the deformation is fully recovered (initial state) (Figure 2c), the separation distance between the negative friction material and the positive friction material will reach a maximum, the positive charge on the surface of the inner electrode of the inner tube and the outer electrode of the outer tube will be completely neutralized, and the shared electrode layer will generate the same amount of induced positive charge. This results in a new electrostatic equilibrium state, and the electrons stop flowing. Subsequently, when the deformation occurs again, the electrons will flow from the inner electrode of the inner tube and the outer electrode of the outer tube to the shared electrode layer in the middle tube (Figure 2d). In the above-mentioned repetitive contact–separation process, an alternating current is generated in the external circuit.

As the result of the flexible hollow inner tube structure, when the structure is deformed, the two triboelectric units form a relatively larger contact area compared with rigid inner tube structure (Figure S2), which can enhance the output performance. Moreover, the flexible hollow structure design makes the whole structure more flexible which makes the human body more comfortable when applied to human motion energy harvesting.

### 3.2. Output Performance of the SNTN

**Figure 3:**
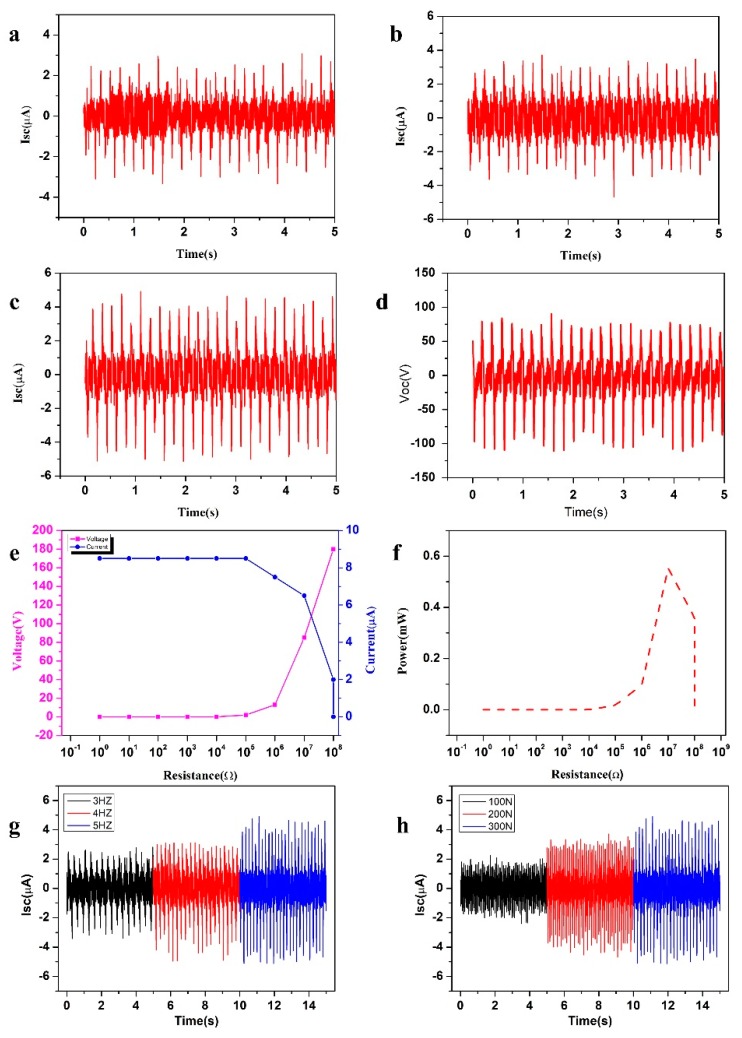
Output performance of the SNTN. (**a**) Short-circuit current of the inner triboelectric unit; (**b**) Short-circuit current of the outer triboelectric unit; (**c**) Short-circuit current of the SNTN; (**d**) Open-circuit voltage of the SNTN; (**e**) Dependence of the voltage and current of the SNTN on the external load resistance; (**f**) Dependence of the power of the SNTN on the external load resistance; (**g**) Short-circuit current of the SNTN with different frequencies; (**h**) Short-circuit current of the SNTN with different forces.

The output performance of the prepared SNTN could be measured systematically by using a linear motor to simulate the press–release process, as shown in Figure S3. According to the power generation mechanism of the SNTN, alternating current is generated in the external circuit. The output short-circuit current and open-circuit voltage used to describe the output performance are both peak-to-peak values which refer to the difference between the highest and lowest values of the signal in a period. With the test pressure and frequency set at 300 N and 5 Hz, respectively, and the length and outer diameter of the SNTN set at 6 cm and 8 mm, respectively, the output performance of the two triboelectric units was tested separately. The short-circuit current of the inner triboelectric unit was 4.5 μA (Figure 3a), the short-circuit current of the outer triboelectric unit was 5.5 μA (Figure 3b), and the total output short-circuit current was 8.5 μA (Figure 3c), which shows that the structure can improve the output performance of the triboelectric nanogenerator. The total output open-circuit voltage was 180 V (Figure 3d). In practical applications, the alternating current (AC) signal can be converted into a direct current (DC) signal by a rectifier circuit (Figure S4), and the negative current can be reversed into a positive current (Figure S5).

Figure 3e displays the output voltage and the output current curve of the SNTN varying with the external load resistance. It can be seen that when the external load resistance is relatively small, the output voltage is almost equal to 0, while the output current is almost equal to the maximum value of 8.5 μA. When the external load resistance is more than 10^5^ Ω, the output voltage increases with increasing load resistance, while the output current decreases with increasing load resistance. When the external load resistance reaches 10^8^ Ω, the output voltage reaches the maximum value of 180 V, while the output current is almost equal to 0. Figure 3f shows that the power increases at first and then decreases, reaching a maximum value of 0.552 mW at a load resistance of about 10^7^ Ω. Here, the power is defined by P = UI, where U and I are the output peak-to-peak voltage and the output peak-to-peak current with different external load resistance, respectively.

Figure 3g shows that the short-circuit current of the SNTN increases with increasing contact–separation movement frequency. When the contact–separation movement frequency provided by the motor increased from 3, to 4, to 5 Hz and the test pressure was 300 N, the short-circuit current increased from 5.5, to 7, to 8.5 µA, respectively. This is because a higher frequency means more contact–separation cycles within a certain time; the contact–separation time becomes shorter, so the same charge could result in a larger current.

In addition, the effective contact distance of the two friction materials in the triboelectric nanogenerator has a significant impact on the output performance: when the effective surface contact distance between the two friction materials reaches molecular spacing [33], it causes the effective charge transfer process of triboelectricity, which plays an important role in enhancing the surface density of friction charges. When a larger external pressure is applied, the friction materials will come into closer contact, the friction charge density will be increased, and the output performance will be enhanced. Figure 3h shows the output short-circuit current of the SNTN with different forces; when the applied force increased from 100, to 200, to 300 N, the short-circuit current increased from 3, to 6, to 8.5 µA, respectively. In this paper, a contact–separation movement frequency of 5 Hz and a force of 300 N were adopted, unless otherwise specified.

**Figure 4:**
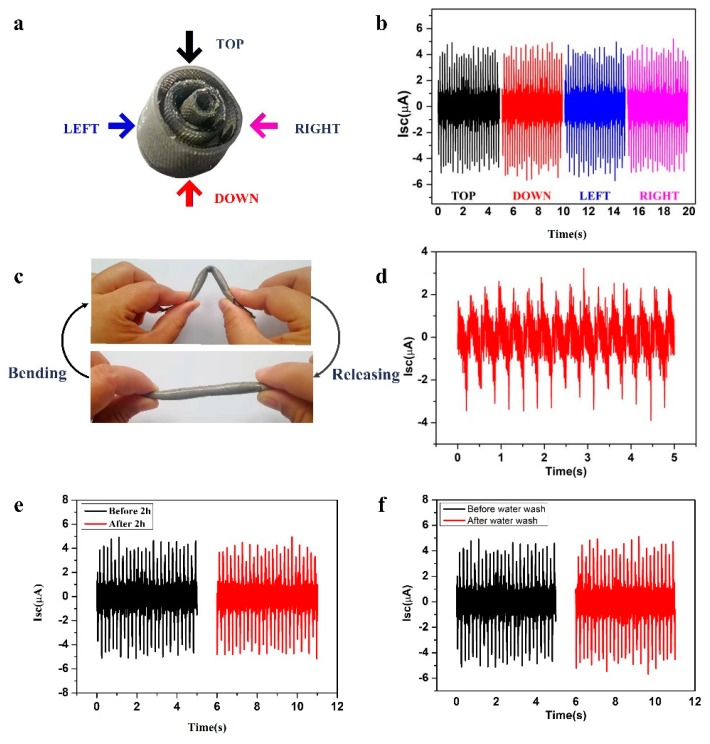
The application performance tests. (**a**) The four different directions perpendicular to the axial direction of the tube; (**b**) Short-circuit current of the SNTN under four pressure directions; (**c**) Schematic diagram showing that the SNTN can collect bending motion energy; (**d**) Short-circuit current of the SNTN when bent by hand; (**e**) Short-circuit current of the SNTN before and after a 2 h pressure process; (**f**) Short-circuit current of the SNTN before and after immersing in water for 2 h.

At the same time, the structure can also harvest external force from different directions perpendicular to the axial direction of the tube, as shown in Figure 4a. When the external force comes from four different directions, the output signal is basically unchanged (Figure 4b), so it is suitable for energy acquisition in different motion directions.

Due to the flexibility of the material selected and the symmetry of the tube structure, the structure can not only harvest pressing motion energy but can also harvest bending motion energy (Figure 4c). The energy generation mechanism of the SNTN is similar to that of a fixed-point press at the bent corner in the bending motion, and a short-circuit current of 5 µA can be obtained when the structure is periodically bent by hand (Figure 4d).

As a power supply instrument for daily electronic equipment, it is especially important for the SNTN to be durable and waterproof. Figure 4e shows the output performance of the SNTN before and after a periodic external force of 300 N and 5 Hz for 2 h. The result indicates that the performance of the SNTN was only slightly decreased. Because the fabricated SNTN is entirely sealed by silicone rubber, which has good hydrophobic properties, its performance is not affected by a humid environment. After the SNTN was immersed in water for 2 h, then the water on the surface removed, the output performance returned to the previous state under the same test conditions (Figure 4f). This result indicates that the SNTN has excellent durability and waterproof performance and can be applied in practical applications.

### 3.3. Applications

**Figure 5:**
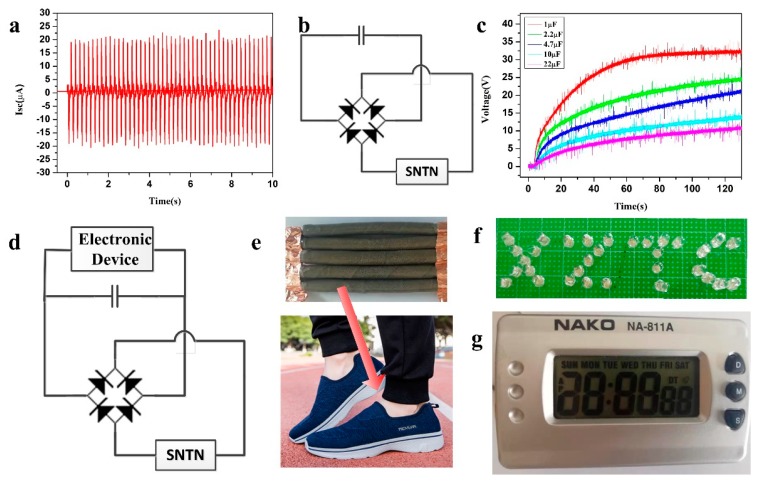
The SNTN as an energy source. (**a**) Short-circuit current of five SNTNs connected in parallel; (**b**) The working circuit of the charging system; (**c**) Charging curves of 1, 2.2, 4.7, 10, and 22 μF capacitors charged by the five SNTNs connected in parallel; (**d**) The working circuit for electronic devices; (**e**) Five SNTNs connected in parallel and fixed under the foot; (**f**) 31 LEDs and (**g**) digital clock applications of the SNTN.

When five prepared SNTNs are connected in parallel, the output short-circuit current can reach 38 µA (Figure 5a). Under external pressure of 300 N and a frequency of 5 Hz, for 1, 2.2, 4.7, 10, and 22 µF capacitors, the voltage can be charged to 10 V within 6, 11, 23, 53, and 103 s (Figure 5b,c). It can be seen that for a larger capacitor, more charging time is needed.

In order to demonstrate the practical applications of the SNTN as an energy harvesting unit, five SNTNs connected in parallel were placed in the heel position to collect the human motion energy during walking and running (Figure 5e). The output electrical energy through normal walking and running was sufficient to light up 31 series LEDs displaying “XZTC” (Figure 5f), as shown in Videos S1 and S2. The 31 LEDs could also be lit up when bent by hand (Video S3). When the output AC signal is converted into a DC signal through a rectifier bridge and then sent to a 2.2 µF capacitor to store energy (Figure 5d), the output electrical energy is sufficient to power up a digital clock (Figure 5g and Video S4). This shows the SNTN’s application prospects in the fields of self-powered lighting and wearable electronic devices.

## 4. Conclusions

In summary, an SNTN was fabricated by nesting a flexible hollow inner tube, hollow middle tube, and hollow outer tube to form an inner triboelectric unit and an outer triboelectric unit, with the two triboelectric units connected in parallel. The parallel structure of the two triboelectric units and the hollow flexible tube structure enhanced the output performance. Moreover, the hollow flexible tube structure is more comfortable for the human body, which fits its potential applications to harvest human motion energy. The fabricated SNTN has the advantages of good flexibility, good biocompatibility, and strong directional applicability, among other. Under an external force of 300 N and 5 Hz, an open-circuit voltage of 180 V and a short-circuit current of 8.5 μA were obtained. When five fabricated SNTNs were connected in parallel and placed under the foot, 31 serially connected LEDs displaying “XZTC” were driven through walking, running, and bending, which shows the SNTN’s application prospects in the fields of self-powered lighting and wearable electronic devices.

## Supplementary Materials

The following are available online at https://www.mdpi.com/2072-666X/10/10/656/s1. Figure S1: SEM image of the Ni-coated polyester conductive textile surface; Figure S2: A contact area comparison of the flexible hollow inner tube structure and a rigid inner tube structure; Figure S3: Schematic diagram illustrating the measurement process using a linear motor to imitate the press–release process; Figure S4: The rectifier bridge circuit; Figure S5: Rectified short-circuit current of the SNTN; Video S1: Supporting video of lighting up 31 commercial LEDs when walking (fixed under the foot); Video S2: Supporting video of lighting up 31 commercial LEDs when running (fixed under the foot); Video S3: Supporting video of lighting up 31 commercial LEDs when bent by hand; Video S4: Supporting video of driving a digital clock when running (fixed under the foot).
