# Supplementary material for: A Shared-Electrode and Nested-Tube Structure Triboelectric Nanogenerator for Motion Energy Harvesting
Source: Micromachines (Basel). 2019 Sep 29;10(10):656. doi: 10.3390/mi10100656 (PMC6843896; doi:10.3390/mi10100656)
Supplement: Supplementary file 1 [file micromachines-10-00656-s001.zip › micromachines-591920 suppl for proofreading/micromachines-591920 suppl for proofreading.docx]

Supplementary Materials: A Shared-electrode and Nested-tube Structure Triboelectric Nanogenerator for Motion Energy Harvesting

Zhumei Tian, Guicheng Shao, Qiong Zhang, Yanan Geng and Xi Chen


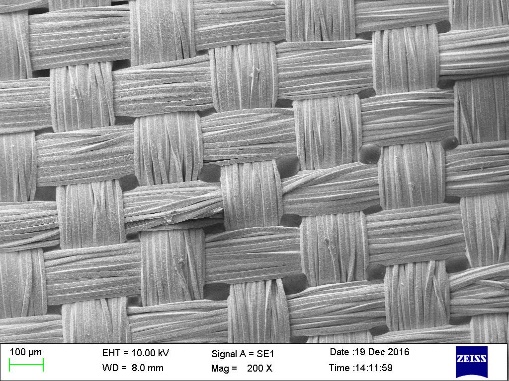


**Figure S1.** SEM image of the Ni-coated polyester conductive textile surface.


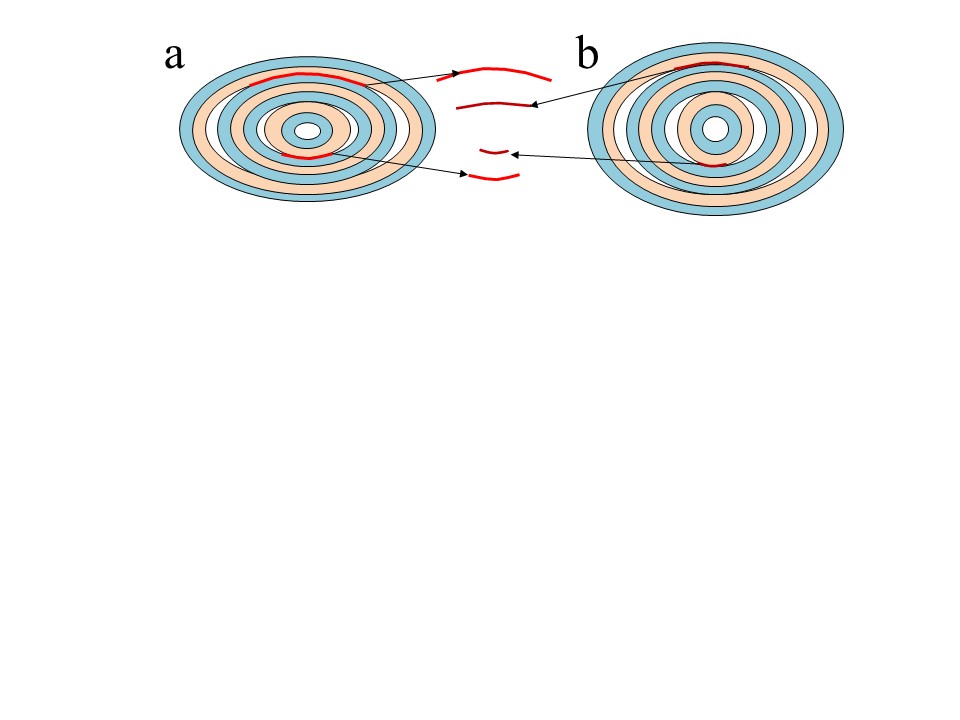


**Figure S2.** A contact area comparison of the flexible hollow inner tube structure and a rigid inner tube structure.


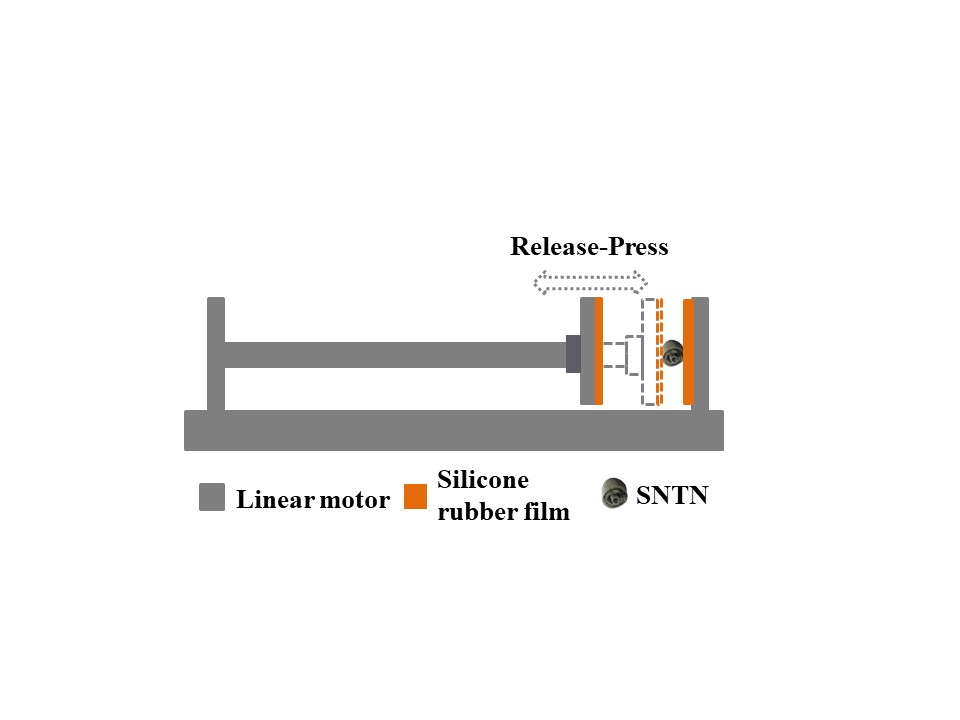


**Figure S3.** Schematic diagram illustrating the measurement process using a linear motor to imitate the press-release process.


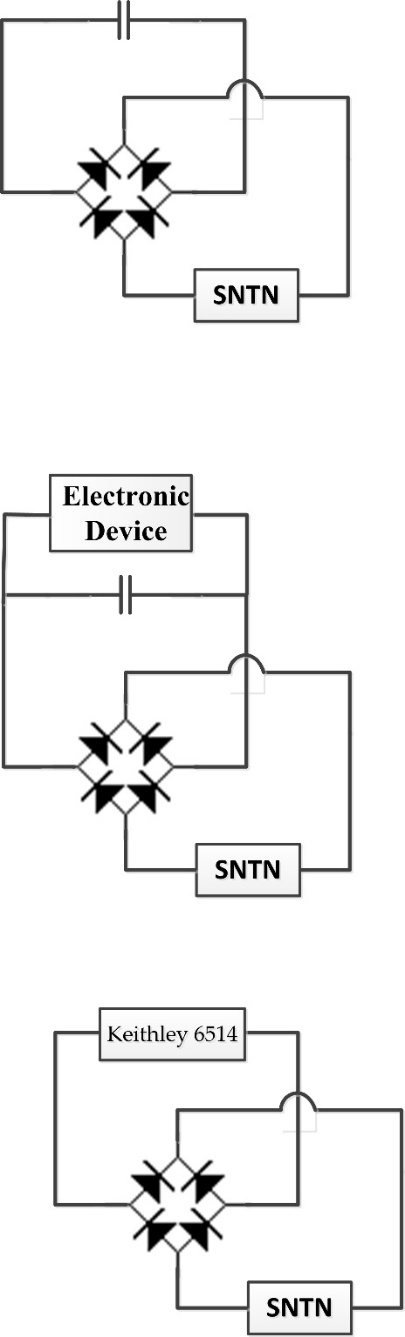


**Figure S4.** The rectifier bridge circuit.


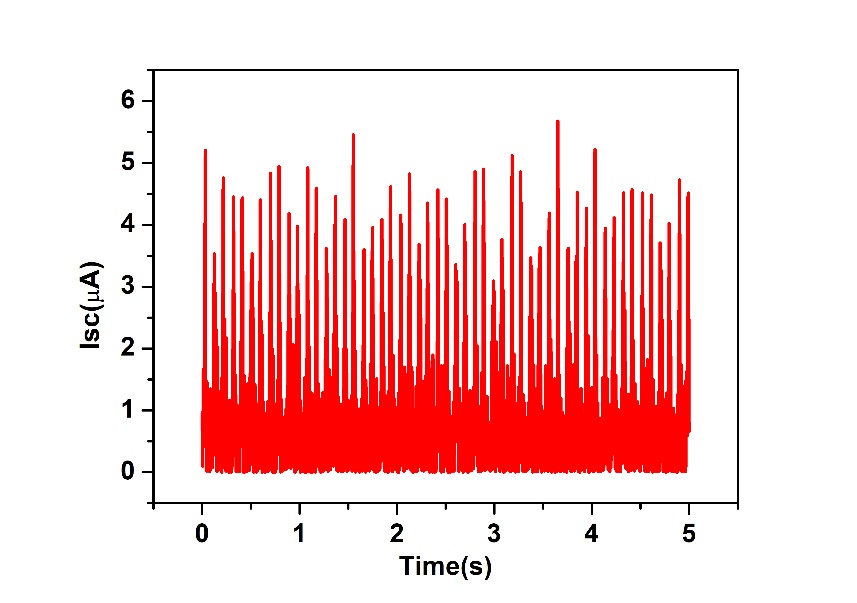


**Figure S5.** Rectified short-circuit current of the SNTN.

**Supporting Videos**

Supporting Video S1: Supporting video of lighting up 31 commercial LEDs when walking (fixed under the foot).

Supporting Video S2: Supporting video of lighting up 31 commercial LEDs when running (fixed under the foot).

Supporting Video S3: Supporting video of lighting up 31 commercial LEDs when bent by hand.

Supporting Video S4: Supporting video of driving a digital clock when running (fixed under the foot).
